# BLE-Based Indoor Localization: Analysis of Some Solutions for Performance Improvement

**DOI:** 10.3390/s24020376

**Published:** 2024-01-08

**Authors:** Filippo Milano, Helbert da Rocha, Marco Laracca, Luigi Ferrigno, António Espírito Santo, José Salvado, Vincenzo Paciello

**Affiliations:** 1 Department of Electrical and Information Engineering, University of Cassino and Southern Lazio, 03043 Cassino, Italy; ferrigno@unicas.it; 2 Department of Electromechanical Engineering, University of Beira Interior, 6200-001 Covilhã, Portugal; helbert.rocha@ubi.pt (H.d.R.); aes@ubi.pt (A.E.S.); jose.salvado@ubi.pt (J.S.); 3 Instituto de Telecomunicações, Delegação da Covilhã, 1049-001 Lisboa, Portugal; 4 Department of Astronautics, Electrical and Energy Engineering, Sapienza University of Rome, 00185 Rome, Italy; marco.laracca@uniroma1.it; 5 Department of Industrial Engineering, University of Salerno, 84084 Fisciano, Italy; vpaciello@unisa.it

**Keywords:** indoor localization, Bluetooth Low Energy, localization solutions

## Abstract

This paper addresses indoor localization using an anchor-based system based on Bluetooth Low Energy (BLE) 5.0 technology, adopting the Received Signal Strength Indicator (RSSI) for the distance estimation. Different solutions have been proposed in the scientific literature to improve the performance of this localization technology, but a detailed performance comparison of these solutions is still missing. The aim of this work is to make an experimental analysis combining different solutions for the performance improvement of BLE-based indoor localization, identifying the most effective one. The considered solutions involve different RSSI signals’ conditioning, the use of anchor–tag distance estimation techniques, as well as approaches for estimating the unknown tag position. An experimental campaign was executed in a complex indoor environment, characterized by the continuous presence in the movement of working staff and numerous obstacles. The exploitation of multichannel transmission using RSSI signal aggregation techniques showed the greater performance improvement of the localization system, reducing the positioning error (from 1.5 m to about 1 m). The other examined solutions have shown a lesser impact in the performance improvement with a decrease or an increase in the positioning errors, depending on the considered combination of the adopted solutions.

## 1. Introduction

Indoor localization has become increasingly important in modern applications, from navigation within large facilities, such as airports and shopping malls [1,2], to quality controls in the industrial field [3]; to use in medicine for therapeutic monitoring [4]; to provide location-based services to enhance the user experience [5,6]; to track and monitor elderly or people with disabilities [7]; to manage the energy consumption based on the occupancy in smart buildings [8]; and to infer occupancy in buildings for crisis management [9]. Indoor localization can be implemented using a variety of technologies, including Wi-Fi, RFID, Ultra-Wideband, and Bluetooth Low Energy (BLE) [10]. Among these technologies, thanks to its specific features, BLE has gained significant popularity in different application fields, including indoor localization. Moreover, the BLE has the advantage of low-power operation compared to Wi-Fi. The location of devices that use the Wi-Fi network requires that the device acquire transmission power from the network in a particular location. The most significant difference to the proposal is that here, it is the device that wants to know its position that works in advertising mode. In this way, energy is only consumed at the time of localization. The BLE was introduced in 2010 as an evolution of classic Bluetooth technology, starting from the version named Bluetooth 4.0, to be energy efficient with a low data rate focused in power constrained and low cost devices [11]. One of the key advantages of using BLE over other indoor localization technologies is its wide availability. BLE technology is integrated into most modern mobile devices, such as smartphones and tablets, making it widely accessible and easily adoptable. A BLE device that is used only for data transmission without receiving data is called a beacon [12]. Beacons are used by large companies, such as Apple, Google, and Facebook, for the development of new standards and products, and are also used by academia and industry for indoor localization, proximity detection, sensing applications, and so on. BLE beacons are inexpensive, small-sized, low-power devices that allow for easy installation and long battery life. In addition to the above-mentioned advantages of BLE technology, it also offers a significant increase in communication range over previous versions [13]. This feature allows for larger areas to be covered with fewer beacon devices, reducing deployment costs and simplifying the management of the considered application. All these specific features of the BLE technology enabled a wide usage in localization systems with an obtainable localization accuracy of about 1 m [14,15].

The localization systems developed using wireless communication technologies and, specifically, the BLE, are composed by means of an anchor-based approach [16]. In detail, the localization system involves the use of some BLE beacons positioned at fixed and known locations (named anchors) and a tag that is a BLE receiver whose unknown position needs to be estimated. For an anchor-based localization system, several quantities can be measured in order to estimate the tag position. For example, approaches based on measures of the Angle of Arrival (AoA), Time of Flight (ToF), and Received Signal Strength Indicator (RSSI) are established in the literature. The AoA-based localization techniques offer high accuracy and is not affected by obstacles or signal reflections [17]. However, it requires expensive and complex hardware to implement, and the coverage is limited by the need for a direct line of sight. The ToF-based techniques provide high accuracy and robustness against interference and noise [18]. However, they require accurate synchronization of anchor clocks and can be affected by reflections and multiple paths, requiring complex algorithms for compensation. The RSSI-based technique is affordable and simple to implement with standard hardware [19]. It has wider coverage and does not require a direct line of sight. However, RSSI has limited accuracy due to environmental variables and can be subject to interference [20,21]. Focusing the attention on the RSSI-based techniques applied to develop localization systems, different solutions have been proposed in the literature to improve the performance of this technology. Many scientific papers propose processing solutions, such as RSSI signal aggregation techniques [14], Kalman filtering of the acquired data [15], transformation models between the RSSI signal and distance [22], and positioning approaches based on optimization algorithms or Machine Learning (ML) [23].

Despite the wide scientific literature proposing different solutions for BLE indoor localization based on RSSI technology, it is still unclear which combination of solutions is the most effective in achieving accurate and reliable localization performance.

The main contributions of this work are listed below.

- Provide a methodology for analyzing different techniques that have the greatest impact on the performance of a BLE-based indoor localization system. In particular, some of the solutions already proposed in the literature are considered and implemented, also considering different combination schemes.
- The analysis was carried out via an experimental campaign in a complex indoor environment characterized by the presence of working staff and numerous obstacles.
- The obtained performance results are compared using different combination schemes of the analyzed solutions in order to identify the solution (or a combination of them) that most contributes to the improvement of a BLE-based indoor localization system.

The performed comparative analysis aims to provide a better understanding of the most effective and reliable solutions for achieving more accurate BLE-based indoor localization. The proposed methodology could help designers of indoor localization systems in identifying which techniques should definitely be used in order to meet the performance requirements of specific applications.

This paper is organized as follows. Section 2 reports a brief description of the used BLE technology, in particular version 5.0, Section 3 discusses the analyzed localization solutions for BLE indoor localization, and Section 4 introduces the experimental set-up used to test the solutions described in Section 3. Finally, the results are shown in Section 5 and the conclusion is provided in Section 6.

## 2. Brief Notes of BLE 5.0 Technology

Bluetooth Low Energy (BLE) technology has become increasingly popular in the context of low-power wireless applications. BLE technology offers 40 transmission channels operating in a 2 MHz frequency band. There are 37 channels for data and 3 channels for advertising (Ch 37, Ch 38, and Ch 39). The introduction of version 5.0 was a significant step in improving the functionality offered by this technology. In this Section, the main benefits and key features of BLE 5.0 technology will be reviewed, with a special focus on its applications in the context of indoor localization. The key advantages of the BLE 5.0 are listed below.

- Reduced energy consumption: BLE 5.0 technology is designed to provide very low power consumption, enabling extended battery life for battery-powered devices. In fact, in BLE, 5.0 devices can have up to 50% lower power consumption than previous versions [13].
- Wide coverage: BLE 5.0 offers a significant increase in range over previous versions. The transmission range can reach up to 200 m in optimal outdoor conditions, enabling wider coverage even in indoor environments by reaching coverage distances of 40 m [13]. This translates into greater flexibility in anchor placement and coverage of larger areas with fewer devices, reducing deployment costs.
- Higher data rates: BLE 5.0 technology supports a data rate of up to 2 Mbps, offering increased information transfer capability. This data rate allows for large amounts of data to be sent efficiently, enabling fast and reliable communication among location devices [13].

BLE devices operating in beacon mode and receiver mode were used in this paper. A BLE beacon is an autonomous transmitter device that sends periodic signals to transmit data to surrounding devices [12]. It operates mainly in advertisement mode and transmits data packets containing information such as beacon identification, transmitted power data, or other useful information. The BLE beacon is generally powered by a battery and has a compact, portable, and low-cost design. BLE beacons are commonly used in indoor localization applications, localization-based marketing, and proximal notifications [24]. On the other hand, a BLE receiver is a device that receives signals transmitted by BLE beacons or other BLE devices [25]. The BLE receiver can detect, decode, and interpret the transmitted BLE data packets. It can be integrated into smartphones, tablets, computers, or other devices capable of supporting BLE connectivity. BLE receivers can detect signals from beacons and use this information for purposes such as localization.

## 3. The Analyzed Localization Solutions

In this paper, we propose an anchor-based positioning system for the tag localization in indoor environments using BLE 5.0 technology. To do this, we use BLE devices operating in beacon mode as system anchors and a BLE receiver as a tag. Details about the implementation of the positioning system are provided in Section 4.

The paper aims to carry out a performance analysis of some solutions, detailed below, adopted in the literature to improve the localization performance typically obtained using BLE technology. In particular, the analyzed solutions cover the processing of the RSSI data (measured by the tag for all anchors to evaluate the tag position), the techniques used to estimate the anchor–tag distances from the RSSI data, and the algorithms used to estimate the tag position. More in detail, in this paper, the analyzed solutions were suitably combined; for each combination, the localization performance was evaluated, and finally, a comparison in terms of localization performance was carried out.

### 3.1. Use of Multichannel Transmission

The BLE technology is characterized by the availability of three advertising transmission channels (Ch 37, Ch 38, and Ch 39) to establish the connection between the BLE devices. The users that develop the BLE-based localization system can define the number of channels to be used for the purpose. On one hand, the use of only one transmission channel allows for simple and low consumption localization systems. From the other hand, the use of multiple transmission channels enable the possibility to make some aggregation strategies to compensate for interference, improving the localization performance (e.g., since the transmission on the three channels takes place with a negligible time interval concerning the tag speed [25], the use of aggregation techniques could reduce the fast-fading effects and interference with Wi-Fi signals [20]). In addition, repetitive measurement of the RSSIs on the transmission channels can be used to both increase the localization capabilities and allow for statistical analysis. Table 1 shows an example of the RSSI values that can be acquired on all three channels, considering N repeated measurements for each channel.

In order to exploit multichannel transmission as a method of improving the performance, in this paper, we compare three different techniques [14] to aggregate the RSSI measured by the three advertising transmission channels as described below.

1. **Maximum technique**
  In this case, the aggregated data coincides with the maximum RSSI measured on the three transmission channels
(1) $$RSSI_{max,i}=\max\left\{RSSI_{37,i},RSSI_{38,i},RSSI_{39,i}\right\},$$
  where *i* denotes the *i*-th repeated measurement. In the rest of the paper, we will refer to this technique as the *max technique*.
2. **Mean technique**
  The second adopted technique aggregates the data, considering the arithmetic average of the measured RSSI on the three transmission channels
(2) $$RSSI_{mean,i}=\sum_{j=37}^{39}\frac{RSSI_{j,i}}{3},$$
  where *i* denotes the *i*- th repeated measurement. In the rest of the paper, we will refer to this technique as the *mean technique*.
3. **Maximum ratio combining technique**
  With the principle of the maximum ratio combining technique, a weighted average is carried out on the RSSI measured by the three transmission channels
(3) $$RSSI_{mrc,i}=\sum_{j=37}^{39}\frac{RSSI_{j,i}-RSSI_{min}}{\sum_{h=37}^{39}RSSI_{h,i}-RSSI_{min}}RSSI_{j,i}$$
  where *i* denotes the *i*- th repeated measurement. Consequently, the channel with the highest (lowest) RSSI will have the highest (lowest) weight in the computation of the aggregated RSSI. The $RSSI_{min}$ value is a constant that represents the receiver sensitivity taken from the datasheet of the adopted BLE device. In the rest of the paper, we will refer to this technique as the *mrc technique*.

Due to the complexity of indoor environments, the measured RSSI data are not reliable due to the presence of obstacles that lead to reflections, attenuation, and multipath effects. To mitigate these problems, data filtering techniques [26] are used to increase the goodness of the RSSI data, allowing us to smooth them from any outliers present in the measurements.

### 3.2. RSSI Filtering

A very important conditioning to be performed on signals before processing them is filtering. In fact, this operation is represented by a transformation function on the spectral structure of the input signal with the purpose of eliminating some unwanted components while leaving others untouched. In the scenario considered in this paper, different types of filters can be used (low-pass, moving average, Savitzky–Golay, etc.). Among the filtering techniques present in the literature, the Kalman filter is considered in the proposed performance analysis since it has proved to be more suitable for solving localization problems also in the static scenario [15,27,28]. The Kalman filter represents a state estimator applicable to a stochastic system. In detail, in the following is reported the stationary linear model in discrete time of the system to be filtered.

(4) $$\left\{\begin{array}{c} x(k+1)=x(k)+w(k)\\ y(k)=x(k)+v(k) \end{array}\right.$$

where

- *k* represents the the discrete time instant;
- *x* represents the filtered RSSI data (state variable);
- *w* represents the process noise;
- *y* represents the measured RSSI data (output variable);
- *v* represents the measurement noise.

The Kalman filter involves the execution of two phases: the prediction and the update. The equations to execute these phases are applied to the stationary linear model reported in (4).

In particular, for the prediction phase in which an estimate of the state variable is carried out based on the system model, we have the following equations:

(5) $$\bar{x}\left(k+1\right)=\hat{x}\left(k\right)$$

(6) $$\bar{P}\left(k+1\right)=P\left(k\right)+R_{w}$$

where $\hat{x}\left(k\right)$ and $\bar{x}\left(k\right)$ represent the *a posteriori* and *a priori* estimate of the state variable, respectively; P(k) and $\bar{P}\left(k\right)$ represent the *a posteriori* and *a priori* state covariance estimate, respectively; and $R_{w}$ represents the process noise covariance matrix.

The update operation that connects the noisy measurements to the state current estimate is described by the measurement update equations:

(7) $$K\left(k\right)=\frac{\bar{P}\left(k\right)}{R_{v}+\bar{P}\left(k\right)}$$

(8) $$\hat{x}\left(k\right)=\bar{x}\left(k\right)+K\left(k\right)\left(y\left(k\right)-\bar{x}\left(k\right)\right)$$

(9) $$P\left(k\right)=\left(1-K\left(k\right)\right)\bar{P}\left(k\right)$$

where K(k) represents the Kalman filter gain and $R_{v}$ represents the measurement noise covariance matrix. The initial values of $\bar{x}\left(0\right)$ and $\bar{P}\left(0\right)$ and the setting values of the $R_{w}$ and $R_{v}$ matrices are provided in Section 5. It is worth noting that the unknown of the problem is represented by $\hat{x}\left(k\right)$ , because it is the state estimate at instant *k* that also uses the measurements at the same instant.

In the localization scenario considered in this paper (static, as will be discussed in subsequent Sections), the Kalman filter is used to estimate noisy constant quantities (RSSI). This represents a simplified case in which to apply the Kalman filter, as it finds its fullest application in dynamic localization scenarios in which a motion dynamics model must be considered.

### 3.3. Distance Estimation

For the estimation of the distances among each anchor and the tag, two different approaches are used in this paper.

- The first approach is made by means of an empirical path loss propagation model based on a logarithmic relationship between the measured RSSI and the distance (10). The estimate of the distance *d* is performed by measuring the RSSI and inverting (10), as shown in (11). The values of *A* and *n* must be estimated during a calibration phase: *A* represents the measured RSSI when d=1 and *n* identifies the path loss exponent.
(10) RSSI=A−10n·log(d)
(11) $$d=10^{\frac{A-RSSI}{10n}}$$
- The second approach is based on a Machine Learning (ML) model, as detailed in the Section 3.4.

The RSSI used for the distance estimation can be the raw measured values or the conditioned ones, applying the aggregation techniques and/or the RSSI filtering described in Section 3.1 and Section 3.2, respectively.

### 3.4. Positioning Approaches

Three different approaches are analyzed in this paper for the estimation of the unknown position: the first is based on numerical optimization, the second uses an ML model integrated with solving an optimization problem, and finally, the last one is completely based on an ML model.

**Figure 1:**
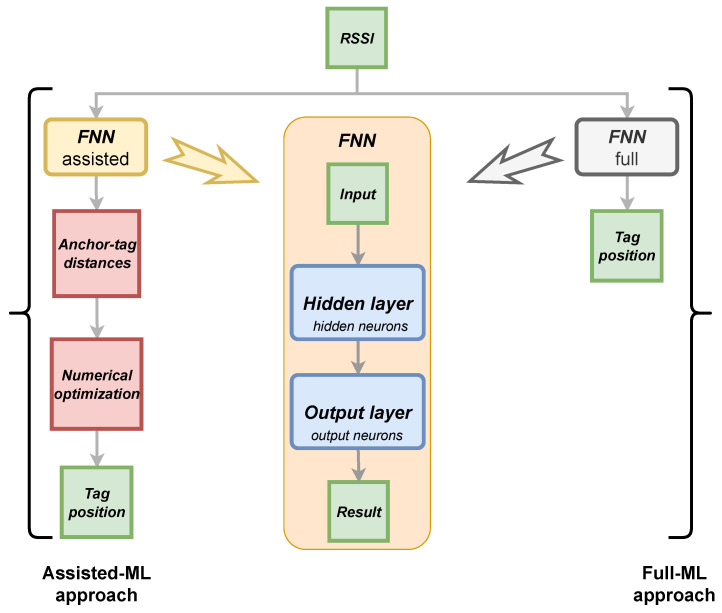
Structure adopted two-layer Feed-Forward Neural Network (FNN) and schematic representations of the Assisted-ML and Full-ML approaches.

1. **Numerical optimization**
  In this case, we define the unknown tag position $\hat{\vartheta }={\left[{\hat{x}}_{t},{\hat{y}}_{t}\right]}^{T}$ as the result of a minimization process defined in (12), where the objective function F(ϑ) is defined in (13).
(12) $$\hat{\vartheta }=\arg\min_{\vartheta }F\left(\vartheta \right)$$
(13) $$F\left(\vartheta \right)=\sum_{i=1}^{M}{\left[{\tilde{d}}_{i}-d_{i}\left(\vartheta \right)\right]}^{2}$$
  In (13), *M* indicates the number of anchors used in the experimental set-up, ${\tilde{d}}_{i}$ indicates the estimated distance between the *i*-th anchor and the tag, while $d_{i}\left(\vartheta \right)$ represents the theoretical distance between the *i*-th anchor and the tag if the tag position is equal to ϑ . Therefore, $\hat{\vartheta }$ is obtained by minimizing the error between the measured and the theoretical quantity for each anchor considered in the experimental set-up. The minimization problem is solved by the Nelder–Mead algorithm [29]. In the rest of the paper, we will refer to this approach as the *numerical optimization approach*.
2. **Assisted-ML approach**
  The ML approaches use a regression model based on a two-layer Feed-Forward Neural Network (FNN). The structure of the adopted FNN is shown in Figure 1 and it was designed in the MATLAB environment. Details about the FNN are given in the following:
  - the input is represented by the aggregate RSSI, obtained with any of the algorithms described in Section 3.1 and filtered; the size of the RSSI vector is equal to the number of anchors installed in the experimental set-up;
  - the hidden layer consists of 20 neurons. It was explored by the authors that this choice represents a good compromise between the computational cost and accurate estimation performance obtained with the FNN considered;
  - the activation functions of the hidden layer and the output layer are sigmoid and linear, respectively;
  - the type of result is represented by the estimated distances between the tag and each anchor used in the experimental set-up; therefore, the number of neurons in the output layer coincides with the number of anchors present in the experimental set-up.
  This approach uses the designed FNN to estimate the RSSI-distance model and, subsequently minimizing an objective function (applying the numerical optimization described above), derives the unknown tag position (see left in Figure 1). Consequently, in the rest of the paper, we will refer to this approach as the *assisted-ML approach*, since only a part of the problem is solved through the use of ML models.
3. **Full-ML approach**
  The FNN described in Figure 1 is used to directly obtain the tag position (see right in Figure 1). Given the RSSI vector as input, it returns the tag position in Cartesian coordinates as a result. Consequently, in this case, the output layer of the FNN consists of two neurons associated with the size of the results (*x* and *y* coordinates).

As described in Figure 1, both ML-based approaches take the aggregated and filtered RSSI signal as input and aim to estimate the unknown position of the tag. To do this, the assisted-ML approach uses FNN to estimate the anchor–tag distances, and then it is necessary to use the numerical optimization approach to estimate the unknown position of the tag. Instead, using the full-ML approach via FNN directly obtains the unknown position of the tag from the input.

## 4. Adopted Experimental Set-Up

This section reports the main features of the adopted BLE devices and provides details on the implementation of the experimental set-up for the tag localization in an indoor environment.

**Figure 2:**
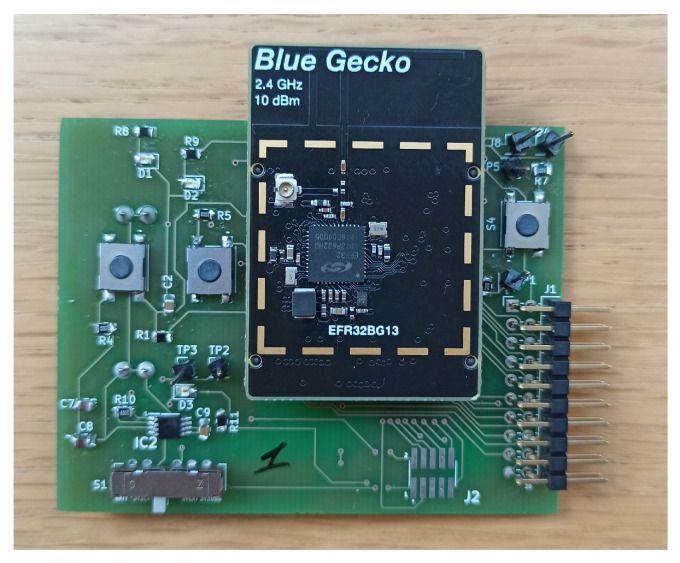
Adopted BLE device with dedicated PCB board.

Blue Gecko Wireless System on Chip (SoC) devices provided by Silicon Labs are used to develop the experimental set-up. Specifically, the devices belong to the EFR32BG13 family [30] and are compatible with the BLE 5.0 standard. Table 2 summarizes the main features of the adopted devices. Furthermore, a Wireless Starter Kit (WSK) mainboard is used to program the devices in the different operating modes, using the Simplicity Studio software (https://www.silabs.com/developers/simplicity-studio, accessed on 1 December 2023). Once the devices have been programmed, to allow for their autonomous operation (without using the WSK mainboard), a suitable PCB board has been designed and realized as shown in Figure 2. In detail, the board has been designed to debug the code loaded on the BLE device, but it also allows for different power supply strategies, including both battery-powered and energy-harvesting systems. Thanks to the PCB board, in previous work [31], the BLE devices used to build the experimental set-up were characterized from an energetic point of view. In the future, the ability to use an energy harvesting system as a power source could remove the problems associated with recharging or replacing batteries.

**Figure 3:**
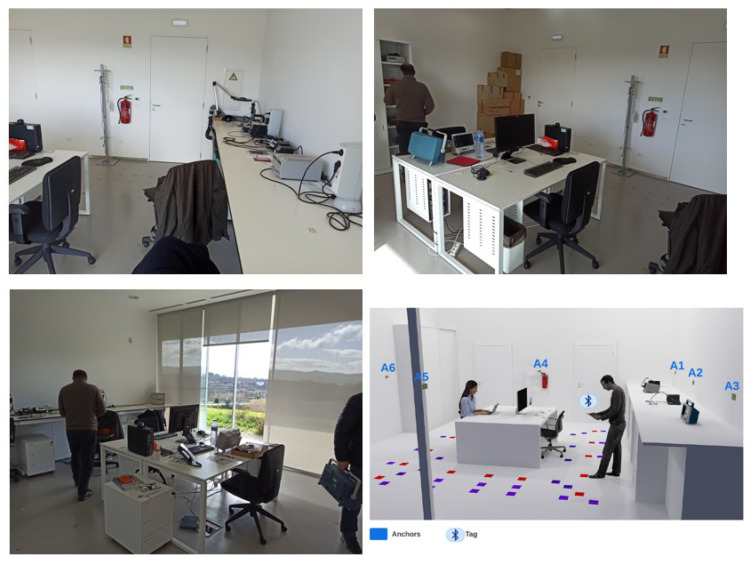
Localization domain inside the Instrumentation and Measurement Laboratory (IML) of the University of Beira Interior, Covilhã, Portugal.

To test the localization solutions to be analyzed in this paper (as described in Section 3), a suitable experimental set-up was developed inside the Instrumentation and Measurement Laboratory (IML) of the University of Beira Interior, Covilhã, Portugal, as shown in Figure 3. The localization domain identified by the laboratory considers an area of about 30 m^2^. The positioning system consists of six anchors placed in a fixed and known position and a tag free to move within the localization domain. The anchors were powered by batteries and their positions within the localization domain are shown in Table 3. The transmission power level considered for the anchors has been set as equal to −16 dBm. The tag was connected to a PC via a USB interface to be powered and to transfer the RSSI measurements sensed from each anchor.

## 5. Obtained Results

In this section, the considered localization solutions are combined and tested via the implemented experimental set-up. It should be noted that the localization domain used to carry out the experimental tests, i.e., IML, constitutes a real scenario in which the typical problems of indoor environments can be encountered. In fact, inside the IML, there are constantly people, interfering signals such as Wi-Fi, obstacles, and walls that can affect the transmission and reception of BLE signals with behavior that is not constant over time.

Some aspects related to the examined procedures and described in Section 3 are initially discussed, also providing numerical details about some parameters considered within the analyzed techniques. Subsequently, the results of localization tests using different combinations of the considered procedures are shown.

### 5.1. Preliminary Considerations

**Figure 4:**
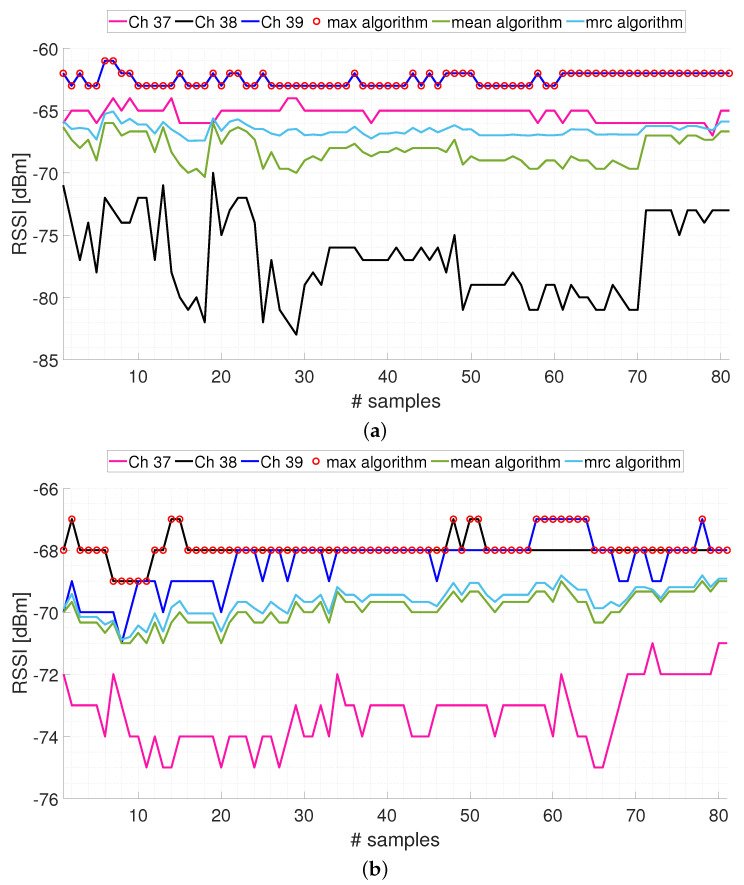
Examples of measured RSSI on the three primary transmission channels (Ch 37, Ch 38, and Ch 39) and adopting the three aggregation techniques (max algorithm, mean algorithm, and mrc algorithm) described in Section 3.1, considering a single anchor-tag pair: (**a**) anchor-tag distance equal to 2.7 m; (**b**) anchor-tag distance equal to 5.2 m.

**Figure 5:**
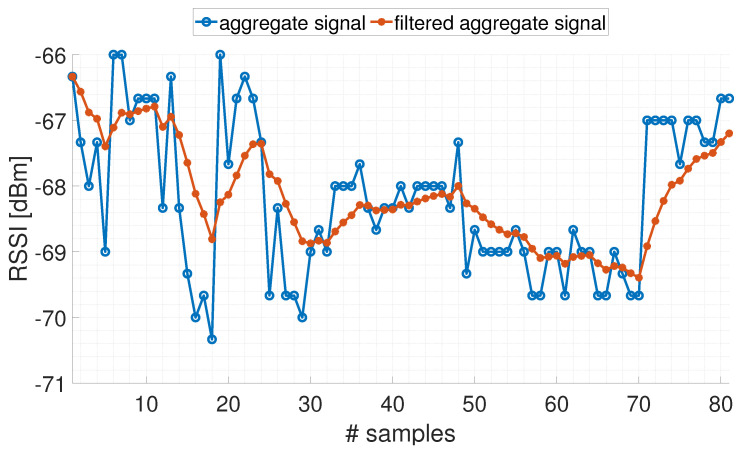
Example of filtering an aggregate signal with mean algorithm.

**Figure 6:**
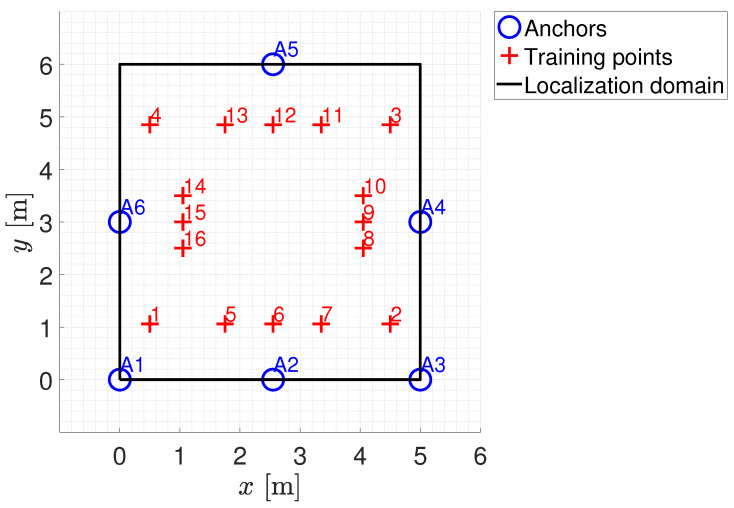
Location of the training points for the calibration phase.

**Figure 7:**
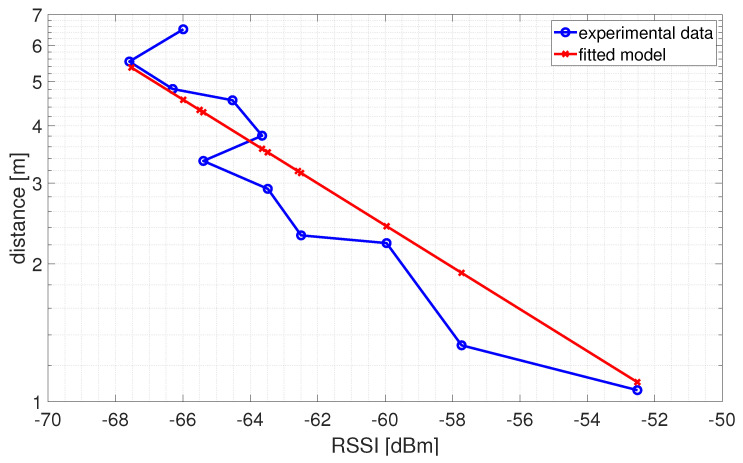
Example of RSSI−distance relationship obtained for anchor A2 and using the RSSI data aggregated with the maximum algorithm and filtered.

**Figure 8:**
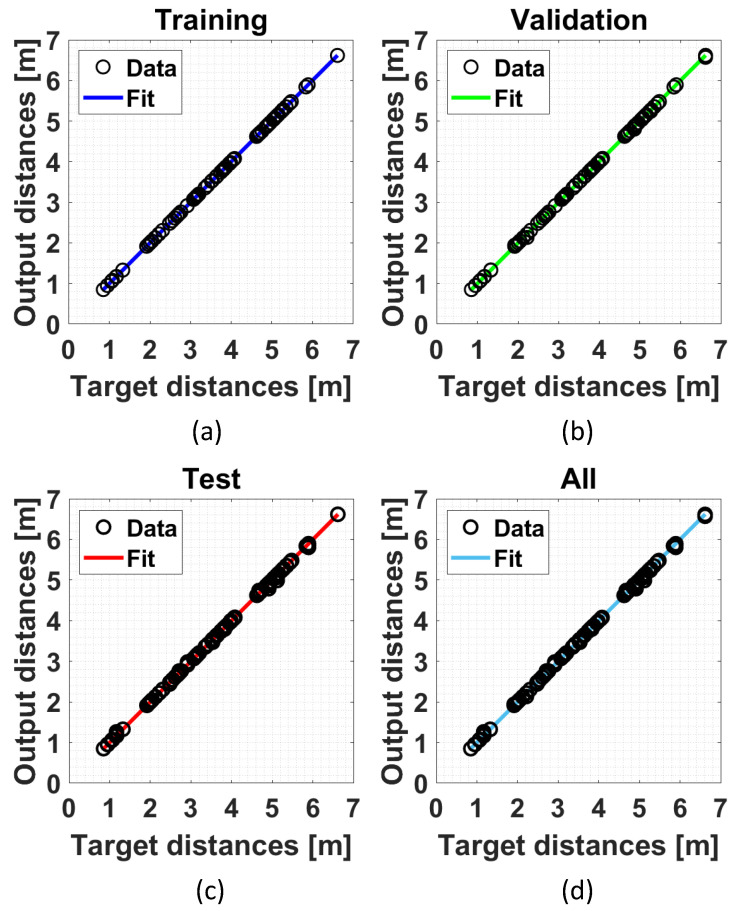
Example of a regression plot obtained for the assisted-ML approach in the following cases: (**a**) training dataset; (**b**) validation dataset; (**c**) test dataset; and (**d**) all datasets.

**Figure 9:**
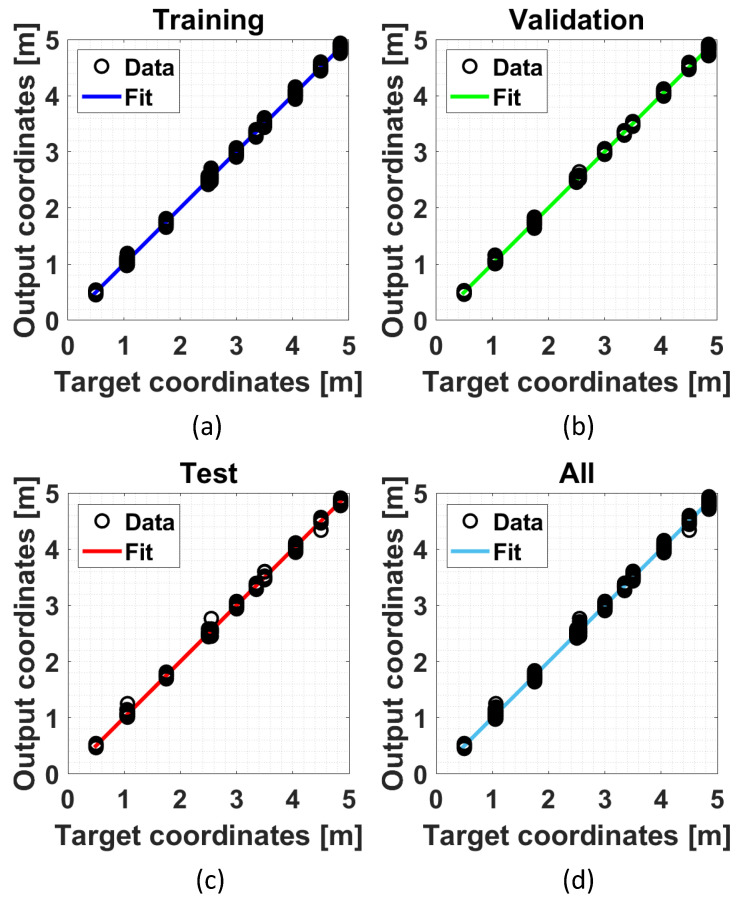
Example of a regression plot obtained for the full-ML approach in the following cases: (**a**) training dataset; (**b**) validation dataset; (**c**) test dataset; and (**d**) all datasets.

1. **Multichannel transmission and aggregation techniques**
  Initially, we want to discuss the use of the analyzed aggregation techniques to exploit multichannel transmission. This represents the first processing operation on the acquired signals within the localization procedures, typically adopted in the literature, considered in this paper. We need to specify the value of the $RSSI_{min}$ parameter in order to compute the mrc algorithm. Based on the datasheet of the considered devices [30], the value of $RSSI_{min}$ is −94 dBm.
  Figure 4 shows an example of the RSSI signals that can be acquired between an anchor and the tag. In particular, the raw RSSI signals acquired on the three primary transmission channels and the aggregate signals obtained with the three aggregation algorithms are shown. The acquisitions were carried out, keeping the anchor and tag stationary, and the considered anchor–tag distances are 2.7 m and 5.2 m for Figure 4a,b, respectively. About eighty acquisition samples were collected. Some considerations are highlighted below.
  - Acquired signals may exhibit high variability. This is shown by the signal Ch 38 in Figure 4a which presents a difference between the maximum and minimum values of 13 dB. Obviously, due to the complexity of indoor environments, this variability makes the localization process inaccurate.
  - The max algorithm sometimes coincides with the signal present on a single channel (as shown in Figure 4a), while other times, it alternates the selection among different channels (as shown in Figure 4b). So, the max algorithm considers more signals if they are close to each other, otherwise it selects only the values of the signal with higher intensity.
  - Unlike the max algorithm, the mean and mrc algorithms always consider the characteristics of all three primary transmission channels. The difference between the two algorithms is that in one case, a simple average is performed (mean algorithm), while on the other hand, a weighted average is performed in which the lower intensity signal has a lower weight than the higher intensity signals (mrc algorithm).
  - The localization process can be affected by the choices made in the number of the adopted transmission channels, by the chosen transmission channels (if less than the available ones), and by the aggregation algorithm to be used for the combination of the RSSI signals.
  In conclusion, the information acquired on the three primary transmission channels contribute, all or in part, depending on the adopted aggregation algorithm, to the formation of an overall aggregate signal to be processed.
  The particular configuration of the room or the building, and the aspects of radio propagation of signal, such as the environment conditions, the obstacles, and the interference due to reflections, have an impact on the level of the signals received. Also, the particular characteristics of the hardware used, namely, the transmitter output power, the sensitivity of the receiver, and the antenna gains, can influence the results. Therefore, by considering another environment configuration (room/building), or using a different hardware setup, can lead to different values of RSSI, although it does not compromise the generality and the applicability of the methodology proposed.
2. **The effect of the filtering procedure**
  As shown in Figure 4a, variability of the acquired signals is present both on the RSSI values coming from the single transmission channel, and on the RSSI values obtained after the application of the aggregation algorithms. The shown variability is the reason for which even if the aggregation algorithms are applied, the RSSI signals are typically filtered. In Figure 5, it is shown an example of the effect of the Kalman filtering approach described in Section 3.2, applied to the signal aggregated by means of the mean aggregation algorithm. The acquisitions were carried out keeping the anchor and tag stationary at a distance equal to 2.7 m. It is noted how all the oscillations present on the aggregate signal are reduced on the filtered signal and how any drift of the aggregate signal in the filtered signal is slowed down. The filtering operation also allows for the discard of possible outliers present in the measurements. As far as the specific settings of the adopted filter, $\bar{x}\left(0\right)$ and $\bar{P}\left(0\right)$ have been set as equal to y(0) (i.e., the first available RSSI measurement) and to 1, respectively; while, via empirical tests, the values of $R_{w}$ and $R_{v}$ have been determined as equal to 1 and 1/20 . This is a common practice when it is necessary to set the parameters of the Kalman filter, as highlighted in [14]. It can be seen that in our procedure, the model has more weight than the measurement process, since the filter is applied to estimate a quantity (RSSI) that is ideally constant.
3. **Calibration and training of the distance/position estimation**
  Some considerations can be made on both the estimation of distances/positions from the RSSI data and the quality of the calibration/training procedures depending on the chosen distance/estimation approaches. As far as distance estimation using the empirical path loss propagation model described in Section 3.3, the RSSI–distance relationship has to be estimated via a preliminary calibration phase. The calibration phase is made by means of an experimental campaign carried out using all the anchors installed in the experimental set-up (see Figure 3). In particular, sixteen training points located within the localization domain were considered. Figure 6 shows the schematic diagram of the identified training points (the training points in the center of the localization domain have not been considered because this area is occupied by a work table, see Figure 3). For each training point, 100 repeated RSSI measurements were performed for each anchor in different times and with environmental conditions. It is important to note that all data used for calibration comes exclusively from experimental tests and there are no data augmentation procedures via numerically simulated training points. In fact, in our opinion, the numerical generation of data, in a complex scenario such as the indoor one where propagation problems are not kept under control, is not reliable given the difficulty in identifying an accurate model for predicting signals.
  RSSI–distance relationships were estimated for each anchor of the experimental set-up using the experimental campaign performed in the calibration phase. In detail, for each anchor, the experimental data were fitted with an analytical model given by (11). Figure 7 shows an example of the result obtained for one of the considered anchors (A2 in Figure 6) using the RSSI data aggregated with the maximum algorithm and filtered. In this example, a Root Mean Square Error (RMSE) of 1.0658 m, a maximum error of 2.3395 m, and a coefficient of determination $R^{2}$ of 0.7224 were obtained on the entire calibration curve shown in Figure 7. The obtained results in terms of the coefficients of the model (11) are equal to A=−51.58 and n=2.18 . By examining Figure 7, it is possible to highlight, as the expected linear behavior of the relationship between the distance and the RSSI (in logarithmic scale) is not perfectly satisfied. This is also confirmed by a low value of the coefficient of determination $R^{2}$ . This is caused by the complexity of the indoor environments with several obstacles and interference signals. The effect is a reduction on the expected accuracy in the tag localization.
  As discussed in Section 3.4, the ML models-based positioning approaches use FNNs (different depending on the specific approach) that need to be trained. The training phase took place with the experimental data obtained during the calibration phase described above. After an arrangement of the collected measurement data, 1600 training points (observations) were obtained for each anchor to be used for the FNN training phase. All available training points have been divided as follows: 70% for training, 15% for validation, and 15% for testing. The FNNs were trained in the MATLAB environment, in which a loss function based on the Mean Squared Error (MSE) was used for training, validation, and testing procedures. Figure 8 and Figure 9 show the obtained results in terms of regression plots for the assisted-ML and full-ML approaches, respectively. Furthermore, Table 4 and Table 5 summarize the results in terms of the used number of observations, MSE, and coefficient of determination $R^{2}$ for training, validation, and testing. The obtained results show good linearity for all used datasets and for both proposed approaches.

### 5.2. Positioning Results

**Figure 10:**
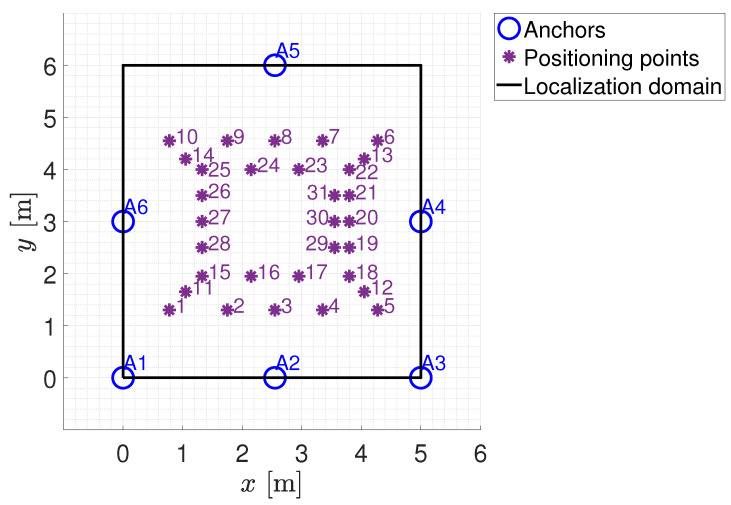
Location of the positioning points for the testing phase.

To verify the localization performance of the considered procedures, a testing phase was carried out. Specifically, as shown in Figure 10, 31 positioning points were considered. For each positioning point, 10 repeated RSSI measurements were collected for each transmission channel, resulting in an available dataset consisting of 930 RSSI measurements for each anchor.

Since the purpose of the paper is to compare the effectiveness of all the considered procedures described in Section 3, the way they were combined is reported below.

- **case #1 –> no aggregation, no filtering**: no conditioning on the acquired signals is carried out. During the calibration phase, the propagation models are estimated for each transmission channel (Ch 37, Ch 38, and Ch 39) using the empirical path loss propagation models. During the testing phase, the obtained propagation models are used to estimate anchor–tag distances, and the estimation of the unknown tag positions is carried out via the numerical optimization approach.
- **case #2 –> no aggregation, yes filtering**: the signals from the transmission channels (Ch 37, Ch 38, and Ch 39) are filtered. During the calibration phase, they are used to estimate the propagation models for each transmission channel (Ch 37, Ch 38, and Ch 39) using the empirical path loss propagation models. During the testing phase, the obtained propagation models are used to estimate anchor–tag distances, and the estimation of the unknown tag positions is carried out via the numerical optimization approach.
- **case #3 –> yes aggregation, no filtering**: the signals from the transmission channels (Ch 37, Ch 38, and Ch 39) are aggregated with all aggregation algorithm (max, mean, and mrc). During the calibration phase, they are used to estimate the propagation models for each aggregation algorithm (max, mean, and mrc) using the empirical path loss propagation models. During the testing phase, the obtained propagation models are used to estimate anchor–tag distances, and the estimation of the unknown tag positions is carried out via the numerical optimization approach.
- **case #4 –> yes aggregation, yes filtering**: the signals from the transmission channels (Ch 37, Ch 38, and Ch 39) are aggregated with all aggregation algorithm (max, mean, and mrc) and filtered. During the calibration phase, they are used to estimate the propagation models for each aggregation algorithm (max, mean, and mrc) using the empirical path loss propagation models. During the testing phase, the obtained propagation models are used to estimate anchor–tag distances, and the estimation of the unknown tag positions is carried out via the numerical optimization approach.
- **case #5 –> assisted-ML**: the signals from the transmission channels (Ch 37, Ch 38, and Ch 39) are aggregated with all aggregation algorithms (max, mean, and mrc) and filtered. During the calibration phase, they are used to train the FNN used in the assisted-ML approach, for each aggregation algorithm (max, mean, and mrc). During the testing phase, the trained FNNs are used to estimate anchor–tag distances, and the estimation of the unknown tag positions is carried out via the numerical optimization approach.
- **case #6 –> full–ML**: the signals from the transmission channels (Ch 37, Ch 38, and Ch 39) are aggregated with all aggregation algorithm (max, mean, and mrc) and filtered. During the calibration phase, they are used to train the FNN used in the full-ML approach, for each aggregation algorithm (max, mean, and mrc). During the testing phase, the trained FNNs are used to directly estimate the unknown tag positions.

All these procedures were applied to the full experimental dataset of unknown tag positions (310 positioning points). To evaluate the performance of each considered procedure (cases #1 to #6), the positioning error ε has been used. It defines the distance between the true and estimated position of the tag, respectively. The mathematical definition is given by (14) where ε represents the Euclidean distance, while ${\left[x_{t},y_{t}\right]}^{T}$ and ${\left[{\hat{x}}_{t},{\hat{y}}_{t}\right]}^{T}$ represent the Cartesian coordinates of the true and estimated position of the tag, respectively.

(14) $$\varepsilon =\sqrt{{\left(x_{t}-{\hat{x}}_{t}\right)}^{2}+{\left(y_{t}-{\hat{y}}_{t}\right)}^{2}}$$

**Figure 11:**
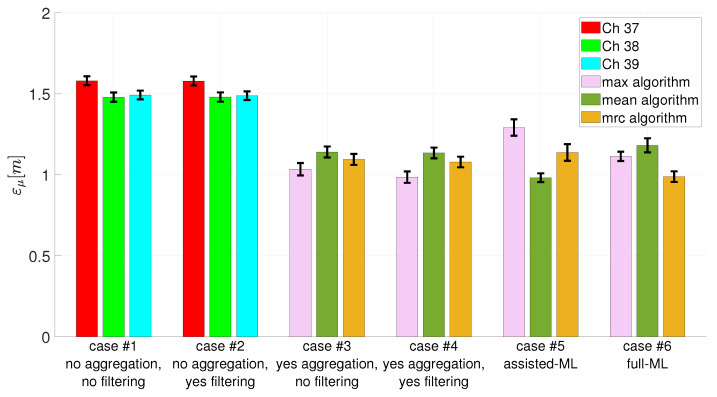
Obtained localization performance in terms of mean positioning errors ( $\varepsilon_{\mu }$ ) and uncertainty on the mean positioning error, for each considered procedures.

Finally, for each considered case, the mean positioning errors ( $\varepsilon_{\mu }$ ) and the corresponding uncertainties on all the analyzed positioning points were estimated. Figure 11 summarizes the obtained results.

In all the considered cases the mean positioning error is always lower than 1.6 m with a maximum uncertainty of 0.06 m. The worst performance, with a mean positioning error of about 1.6 m, are obtained in the first two analyzed cases, case #1 and case #2. In both these cases, multichannel transmission was not exploited since no aggregation algorithm was applied. The filtering effect does not allow any localization improvement since the performances are very similar between case #1 and case #2. In addition, it is possible to highlight, as the chosen transmission channel does not have any significant effect on the localization performance. Regarding the other analyzed cases, we obtain consistently better performance compared with case #1 and case #2 (about 1.0 m as the mean positioning error). The key contribution to achieve the performance improvement is related to the use of the aggregation algorithms. In fact, all four cases (from case #3 to case #6) apply the three considered aggregation algorithms. As far as the used aggregation algorithm, there is no trend towards improvement. In some cases, it is better to use the max algorithm (cases #3 and #4); in others, the mean or the mrc algorithm, depending on the used ML approach. Regarding the comparison among the considered positioning procedures, it is necessary to refer to cases #4, #5, and #6 as they share the same operating conditions (RSSI measurements are aggregated with all the considered aggregation algorithms and filtered). Having fixed the operating conditions, in case #4, the empirical path loss propagation model is used to estimate the anchor–tag distances and then the numerical optimization algorithm is used to estimate the unknown tag positions. In case #5, the anchor–tag distances are estimated via the FNN employed in the ML-assisted approach, and then via the numerical optimization algorithm, the unknown tag positions were estimated. Finally, in case #6, via the FNN employed in the full-ML approach, the unknown tag position were directly estimated from the RSSI measurements. Using the mean as the aggregation algorithm, the assisted-ML approach guarantees the best performance compared to the previous ones, while it exhibits worse performance using max and mrc as aggregation algorithms. Of course, these considerations are valid with respect to the adopted conditions of algorithm setting and the considered testing scenario. Anyway, a limited performance variation (mean positioning error) can be observed. In all the considered cases (cases from #1 to #6), a limited variation in the uncertainty of the mean positioning error was observed, not allowing any relation with the chosen procedures.

### 5.3. Discussion

The proposed analysis highlighted as the localization performance using BLE technologies are strictly connected on the use of multiple transmission channels that enable the application of aggregation algorithms. If a worse performance is acceptable, it is possible to apply a single transmission channel, and no suitable choices on the transmission channel to be used have to be made. If a better performance is needed, the multiple transmission channel must be enabled and the choice of the positioning algorithms can be substantially made, considering the minimum computational cost. The suggested methodology may assist indoor localization system designers in determining which solutions must be employed to satisfy the demands of particular applications in terms of performance.

Certainly, several activities can be carried out in the future to extend the methodology proposed in this paper. For example, a more exhaustive analysis concerns the execution of the experimental campaign in larger working environments, or varying the density and the geometric placement of beacons within the localization domain and verifying whether the claimed best solutions remain so as these conditions vary. Other important activities include conducting experimental tests in a dynamic localization context and testing the generality of the considered techniques (especially ML-based approaches) by considering greater variability in the data used for both training and testing. Such variability could come from experimental campaigns carried out on different working environments. In addition, it is possible to consider improved versions of the considered and implemented solutions (e.g., regarding the propagation model or neural network), but also to add other techniques (e.g., regarding filtering or positioning) which are not considered in this paper.

## 6. Conclusions

In the framework of the localization techniques based on Bluetooth Low Energy (BLE) 5.0 technology using the Received Signal Strength Indicator (RSSI), different solutions have been proposed in the literature to improve the localization performance. An experimental campaign in a complex indoor environment was carried out to identify the solution (or a combination of them) that most contributes to the improvement of BLE-based indoor localization systems. The obtained results showed that the exploitation of multichannel transmission through the use of RSSI signal aggregation techniques is the most crucial aspect for achieving optimal performance. The multichannel approach, based on combining RSSI signals from the different transmission channels (Ch 37, Ch 38, and Ch 39), takes full advantage of the potential of BLE 5.0 technology, allowing us to reduce the positioning error of about 35% (from 1.5 m to 1 m). Other solutions have been considered and analyzed, also in combination among them: the RSSI signal filtering; distance estimation adopting an empirical propagation model or Machine Learning (ML); numerical optimization; and ML models for estimating the unknown position of the tag. These solutions have showed a lesser impact in the improvement of the localization accuracy with an increase or a decrease in the positioning error that goes from 2% to 23%, depending on the combination of the used solutions. The results of this analysis can be useful for the designers to choose the solutions to be implemented, depending on the target accuracy of the localization system to be developed. Certainly, the obtained results have full validity for the conditions tested and considered in this paper, but they might be subject to variations considering other operating conditions.

## Figures and Tables

**Table 1:** RSSI measured by the tag for each anchor.

Samples	Ch 37	Ch 38	Ch 39
1	$RSSI_{37,1}$	$RSSI_{38,1}$	$RSSI_{39,1}$
2	$RSSI_{37,2}$	$RSSI_{38,2}$	$RSSI_{39,2}$
⋮	⋮	⋮	⋮
*i*	$RSSI_{37,i}$	$RSSI_{38,i}$	$RSSI_{39,i}$
⋮	⋮	⋮	⋮
*N*	$RSSI_{37,N}$	$RSSI_{38,N}$	$RSSI_{39,N}$

**Table 2:** Main features of the adopted Bluetooth devices.

Device code	EFR32BG13P632F512GM48-D
Supported protocol	BLE 5.0
Operating band	2402–2480 MHz
Maximum transmit power	+10 dBm
Sensitivity ( $RSSI_{min}$ )	−94 dBm
Integrated antenna	Printed inverted–F
MCU	32–bit 40 MHz ARM Cortex–M4
Flash memory	512 kB
RAM	64 kB
Main integrated functionality	Debug and packet trace; Advanced Energy Monitoring; Virtual COM Port

**Table 3:** Positions of the anchors inside the localization domain.

Anchors	*x* [m]	*y* [m]
A1	0	0
A2	2.5	0
A3	5	0
A4	5	3
A5	2.5	6
A6	0	3

**Table 4:** Training results for each dataset for the assisted-ML approach.

	Observation	MSE [m^2^]	R^2^
Training	1098	4.3666 × 10^7^	1.0000
Validation	235	1.1190 × 10^−5^	1.0000
Test	235	6.1233 × 10^−5^	1.0000

**Table 5:** Training results for each dataset for the full-ML approach.

	Observation	MSE [m^2^]	R^2^
Training	1098	4.9483 × 10^−4^	0.9999
Validation	235	8.3280 × 10^−4^	0.9998
Test	235	9.0197 × 10^−4^	0.9998

## Data Availability

Data are contained within the article.
